# Sex Differences in Social Interaction Behavior Following Social Defeat Stress in the Monogamous California Mouse (*Peromyscus californicus*)

**DOI:** 10.1371/journal.pone.0017405

**Published:** 2011-02-25

**Authors:** Brian C. Trainor, Michael C. Pride, Rosalina Villalon Landeros, Nicholas W. Knoblauch, Elizabeth Y. Takahashi, Andrea L. Silva, Katie K. Crean

**Affiliations:** Department of Psychology, University of California Davis, Davis, California, United States of America; University of Minnesota, United States of America

## Abstract

Stressful life experiences are known to be a precipitating factor for many mental disorders. The social defeat model induces behavioral responses in rodents (e.g. reduced social interaction) that are similar to behavioral patterns associated with mood disorders. The model has contributed to the discovery of novel mechanisms regulating behavioral responses to stress, but its utility has been largely limited to males. This is disadvantageous because most mood disorders have a higher incidence in women versus men. Male and female California mice (*Peromyscus californicus*) aggressively defend territories, which allowed us to observe the effects of social defeat in both sexes. In two experiments, mice were exposed to three social defeat or control episodes. Mice were then behaviorally phenotyped, and indirect markers of brain activity and corticosterone responses to a novel social stimulus were assessed. Sex differences in behavioral responses to social stress were long lasting (4 wks). Social defeat reduced social interaction responses in females but not males. In females, social defeat induced an increase in the number of phosphorylated CREB positive cells in the nucleus accumbens shell after exposure to a novel social stimulus. This effect of defeat was not observed in males. The effects of defeat in females were limited to social contexts, as there were no differences in exploratory behavior in the open field or light-dark box test. These data suggest that California mice could be a useful model for studying sex differences in behavioral responses to stress, particularly in neurobiological mechanisms that are involved with the regulation of social behavior.

## Introduction

Stressful life experiences are known to contribute to the development of mood disorders [1], yet the mechanisms that translate these stressful experiences into behavior are poorly understood. The development of imaging techniques to assess changes in neurobiological activity associated with mood disorders has provided new insights [2], but these approaches can not establish cause and effect relationships. Some behavioral symptoms of mood disorders can be modeled in non-human animals, which allows for the use of experimental approaches that can identify causal mechanisms [3]. The social defeat model induces a constellation of long lasting behavioral responses in many species that mimic aspects of psychiatric disorders [4]. The rationale for this model is that social conflict can be a precipitating factor for many mood disorders [5], [6]. One of the most robust responses to social defeat is reduced social interaction behavior, which is manifested by reduced social investigation (mice and rats) [7], [8], [9], reduced sexual behavior (mice and tree shrews)[10], [11] and reduced territorial aggression (Syrian hamsters)[12], [13]. The reduction in social interaction can be reversed with chronic, but not acute antidepressant treatments [8]. This is significant because it matches the timeline of human behavioral responses to antidepressants. The most commonly studied laboratory rodents have low levels of female-female aggression, so it has been difficult to study female responses to defeat (but see [14]). This is problematic because depression and anxiety disorders are more common in women than men [15], [16]. Here we describe data collected from the monogamous California mouse (*Peromyscus californicus*), in which the social defeat paradigm is examined in both males and females.

The California mouse is unique because males and females aggressively defend joint territories against same sex intruders [17]. These aggressive behaviors can be observed during resident-intruder tests in a laboratory setting, thus allowing for the use of the social defeat model in an ethologically relevant context. We previously observed that female, but not male, California mice show increased glucocorticoid secretion following aggressive interactions [18]. In some cases, exaggerated glucocorticoid responses are associated with increased risk of mental disorders [19]. Based on these data we hypothesized that females would show stronger behavioral responses to social defeat than males. Mice exposed to episodes of defeat or control episodes were behaviorally phenotyped in tests assessing social interaction behavior and exploratory behavior. Blood samples were collected to assess sex differences and effects of defeat on corticosterone levels. We examined phosphorylated CREB (pCREB) and phosphorylated extracellular-signal regulated kinase (pERK) expression in the “extended amygdala” as indirect markers of cellular activity. Anatomical and functional similarities among the amygdala, bed nucleus of the stria terminalis (BNST), and nucleus accumbens (NAc) shell have led some research groups to refer to these regions as the “extended amygdala” [20]. Nuclei within this circuit are known to mediate behavioral responses induced by social defeat [8], [21], [22], so we examined indirect markers of brain activity in these nuclei following social interaction testing. Phosphorylation of ERK and CREB can be induced by several different cellular pathways [23], [24], [25] and so analyses of these proteins provides a coarse measure of cellular activity. Activation of ERK (pERK) can reflect changes in intracellular calcium and the activity of tyrosine kinase receptors. Once phosphorylated, ERK can alter neuronal excitability [26] and increase phosphorylation of CREB. CREB is a broader marker because it can also be phosphorylated by changes in cAMP, which is regulated in part by G protein receptors. Our results demonstrate sex differences in behavioral responses to social defeat that are long lasting and context dependent. We also present data describing the divergent effects of social defeat stress on glucocorticoids and brain responses in males and females.

## Methods

### Animals

California mice (*Peromyscus californicus*) were bred in our laboratory colony at UC Davis. Mice were individually marked with ear punches and housed in clear polypropylene cages provided with Carefresh bedding and cotton nestlets. Harlan Teklad 2016 food and water were provided *ad libitum*. Mice were maintained on 16 h light/8 h dark cycle (lights off 1400 PST), a summer-like light cycle that is commonly used in studies of *Peromyscus* [27], [28]. Although this summer-like light cycle is required for many species of *Peromyscus* to be reproductively active [29], the reproductive system in California mice is not suppressed under winter-like short day light cycles [27], [30], [31], [32]. All testing procedures were approved by the UC Davis Institutional Animal Care and Use Committee (Protocol 15425). Unless otherwise indicated, all mice were 3 month old adults and housed 2–3 per cage in same sex groups. Behavioral observations were conducted in the dark phase under dim red light (3 lux) except for the light-dark box test which was conducted during the light phase (150 lux). Animals were maintained in accordance with the recommendations of the *National Institutes of Health Guide for the Care and Use of Laboratory Animals*.

### Experiment 1

#### General experimental design

In experiment 1 mice were randomly assigned to be exposed to social defeat or control episodes. Males and females assigned to social defeat were exposed to highly aggressive same-sex breeders on three consecutive days (n = 9 males, n = 11 females). Although virgin male and female California mice exhibit aggressive behavior [33], [34], pilot studies indicated that there was less variability in aggression levels among breeders than virgin mice. Pups and the breeder's mate were removed 5 min before each episode of defeat, which lasted 7 minutes or until the breeder attacked the focal mouse 10 times. Control mice were introduced into a clean cage for 7 minutes and then returned to the home cage (n = 10 males, n = 15 females). This paradigm is similar to studies conducted in rats, but milder than studies on domestic mice which use continual sensory contact with an aggressive resident [8], [35]. After exposure to defeat, each focal mouse was returned to its home cage and cagemates. We hypothesized that three episodes of defeat would be salient because previous work demonstrates that three winning experiences has important effects on brain and behavior [36], [37].

A complicating factor when studying intact females is accounting for variation in the ovarian cycle. Ideally, we would have used vaginal lavage before behavioral tests to identify females in diestrus, proestrus, or estrus before beginning social defeat or control training. However, lavage itself is stressful and has significant effects on behavior in female California mice ([30]; E. S. Davis personal communication). Because social defeat and control training was conducted across multiple days, each female was trained across multiple stages of the estrous cycle. Thus although variation in estrous cycle could influence the severity of each episode of defeat, multiple bouts of defeat ensured that this variation was not a confounding factor.

#### Social interaction test

**Figure 1:**
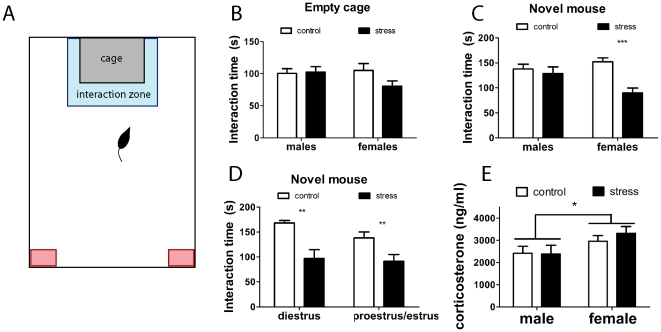
Social interaction behavior in male and female California mice four weeks after social defeat. In the apparatus used for testing (A), the interaction zone is indicated by a blue box and the corner zones indicated by red boxes. There were no significant differences when mice were tested with an empty cage (B). Females, but not males exposed to social defeat showed reduced social interaction behavior with a novel mouse (C). Immediately after the social interaction test mice were euthanized and vaginal lavage was conducted to determine estrous cycle stage. Social defeat reduced social interaction time at different stages of the estrous cycle (D). Corticosterone levels measured immediately after social interaction testing were higher in females compared to males (E). * main effect of sex p<0.05, ** planned comparison control group versus stress group p<0.01. *** planned comparison control group versus stress group p<0.01. All data are mean±s.e.

Social interaction behavior was investigated using an apparatus consisting of a large open field (Fig. 1a, 89×63×60 cm) containing a small wire cage (14×17×14.5 cm). Each focal mouse was introduced into the open field for 3 min to habituate, and we recorded the amount of time the focal mouse spent interacting with the empty wire cage (within 8 cm, see blue box in Fig. 1A) using a video tracking system (Stoelting, Wood Dale, IL). Next an unfamiliar, same sex virgin mouse was introduced into the wire cage. For 3 min we recorded the amount of time the focal mouse spent interacting with the wire cage. We also measured time spent in the two corners opposite the wire cage (8×8 cm, Fig 1A) and total distance traveled as an estimate of total activity. After each test the arena was cleaned with 70% ethanol and dried before the next mouse was tested. Social interaction was assessed at 24 hours and 4 weeks after social defeat exposure. Different stimulus mice were used for the two tests. In between the two social interaction tests the mice were undisturbed except for routine cage changes. Immediately after testing at 4 weeks, each focal mouse was anesthetized with isoflurane and euthanized by decapitation (14:45–17:00 PST). Brains were collected immediately after testing to detect changes in phosphorylated CREB and ERK, which we have previously quantified in California mice after 7 min resident-intruder tests [30], [38]. Trunk blood was collected in heparinized tubes and centrifuged to collect plasma (see below for corticosterone assay methods). Brains were quickly removed and immersion fixed in 5% acrolein in phosphate buffered saline (PBS). Each female was lavaged post-mortem. Estrous cycle stage was determined by assessing the presence of leukocytes, nucleated epithelial cells, and/or cornified cells [30], [39].

#### Immunohistochemistry and Quantification

Brains were sectioned at 40 µm on a microtome and stored in cryoprotectant (50% v/v phosphate buffer, 30% w/v sucrose, 1% w/v polyvinylpyrrolidone, 30% v/v ethylene glycol) at −20°C. Sections were then washed 3 times in PBS and incubated in 1% sodium borohydride in PBS for 10 min. Sections were then blocked in 10% normal goat serum and 0.3% hydrogen peroxide in PBS for 20 min. Sections were then incubated in primary pCREB (Cell Signaling, 1∶100) or pERK (Cell Signaling, 1∶250) antibodies dissolved in 2% normal goat serum and 0.5% triton X (TX) in PBS overnight at 4°C on an orbital shaker. These primary antibodies have been used previously in California mice [38]. The sections were then washed three times in PBS before transferring to biotinylated goat anti-rabbit antibody in 2% normal goat serum in PBS TX (Vector Laboratories, Burlingame, CA, 1∶500) for 2 hr. Sections were washed 3 times in PBS and incubated in avidin-biotin complex (ABC Elite Kit, Vector Laboratories) for 30 min. Sections were then washed 3 times in PBS and developed in nickel enhanced diaminobenzidine (Vector Laboratories) for 2 minutes. Sections were then rinsed in PBS and mounted onto plus slides (Fisher, Pittsburgh, PA). Slides stained for pERK were dehydrated in ethanol followed by Histoclear (National Diagnostics, Atlanta GA) and coverslipped with Permount (Fisher). Slides stained for pCREB were dehydrated, counterstained with eosin, cleared with Histoclear, and coverslipped with Permount.

**Figure 2:**
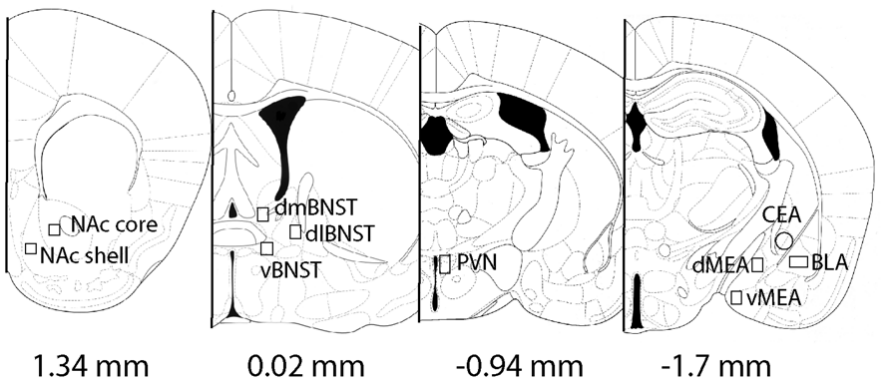
Representation of areas quantified using microscopic analyses in experiment 1. Reproduced from Paxinos & Franklin (2003), with permission from Academic Press. Abbreviations: Nucleus accumbens (NAc), dorsomedial bed nucleus of the stria terminalis (dmBNST), dorsolateral bed nucleus of the stria terminalis (dlBNST), ventral bed nucleus of the stria terminalis (vBNST), paraventricular nucleus (PVN), basolateral amygdala (BLA), central nucleus of the amygdala (CEA), dorsomedial amygdala (dMEA), dorsoventral amygdala (vMEA).

Representative photomicrographs (Fig. 2) were taken with a Zeiss AxioImager and were based on a mouse brain atlas [40]. The background for each image was normalized by adjusting the exposure time. The number of immunopositive cells in each brain area was counted in a frame of uniform size (NAc core, 0.3×0.29 mm; NAc shell, 0.3×0.29 mm; dorsomedial BNST, 0.53×0.3 mm; dorsolateral BNST, 0.53×0.33 mm; ventral BNST, 0.34×0.34 mm; PVN 0.24×0.18 mm; dorsal MEA, 0.33×0.38 mm; ventral MEA, 0.33×0.38 mm; BLA, 0.38×0.19 mm; CEA, 0.38 mm diameter circle) using Image J (NIH, Bethesda, MD) by an observer unaware of treatment assignments. The number of positive cells was counted using the “analyze particles” function of Image J. Cell count data are presented as number of positive cells per mm^2^.

#### Data Analysis

Analyses of Q-Q plots indicated that the data from social interaction tests were normally distributed, and variances were homogenous across treatment groups. We used two-way ANOVA testing for effects of stress and sex to analyze time spent interacting with the empty cage and the novel target mouse. Separate analyses were conducted using data from the tests conducted at 24 hr and 4 weeks after the last episode of social defeat. An additional two-way ANOVA was performed on the data from the 4 week test, this time comparing males, diestrus females, and proestrus/estrus females. These categories were based on the results of postmortem vaginal lavage observations. Although we previously observed that estradiol levels are elevated in proestrus females [30], we combined proestrus and estrus females for this analysis because there were too few females in these states to analyze separately. This may be explained by previous observations showing that the diestrus phase in California mice is variable and can be considerably longer than rats or mice [41]. Analyses of Q-Q plots indicated that corticosterone and immunohistochemistry cell count data were not normally distributed, so Mann-Whitney U-tests were used to analyze these data. We also used Spearman rank correlations to examine relationships between cell counts across brain areas and with behavior.

### Experiment 2

#### General experimental design

**Figure 3:**
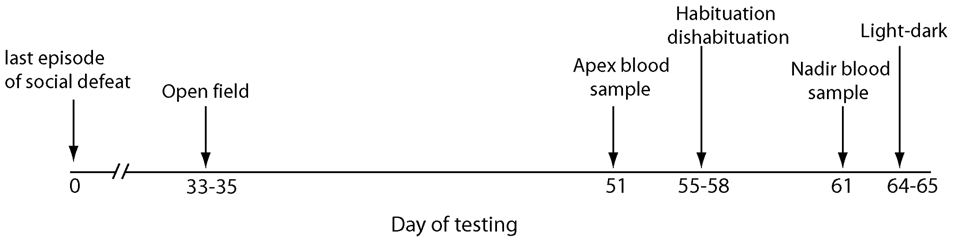
Timeline of procedures in Experiment 2.

In a second set of mice (See Fig. 3 for timeline), males and females were randomly assigned to defeat (n = 9 males, n = 9 females) or control conditions (n = 10 males, n = 10 females) as described for experiment 1. The number of offensive attacks was quantified during each episode of defeat. Starting 4 weeks after the last social defeat test, mice were tested in open field (days 33–35), habituation-dishabituation tests (days 55–58), and light-dark box tests (days 64–65). We attempted to minimize the effect of experience of the different behavioral tests by waiting about 1 week in between each test [42] and several days after blood sampling. On day 51 following social defeat, retroorbital blood samples were collected 30 min after lights out (apex sample, 1430–17:00 PST). On day 61, a second retroorbital blood sample was collected 5 hrs before lights out (nadir sample, 08:00–09:30 PST). Each cage was removed one at a time from the colony room to an adjacent procedure room, and each mouse was anesthetized with isoflurane for 60 sec before a blood sample was collected. All samples were taken in quick succession and mice were placed in a clean cage immediately after sampling to avoid exposing cagemates to blood. All cages were returned to the colony room after the last blood sample was collected. Light-dark box tests were conducted on days 64–65. Body weights were taken on day 55 and a sucrose preference test was conducted on day 42 (data not shown). We did not monitor estrous cycles during the study because conducting vaginal lavage in California mice has large effects on female behavior [30].

#### Behavioral testing

Our tests of social interaction in experiment 1 were conducted in a novel environment, which can induce anxiety-like behavior in rodents [43]. To assess whether social defeat alters responses to social stimuli in a familiar environment, we conducted habituation-dishabituation tests within the home cage. Habituation-dishabituation tests consisted of 9 consecutive 2 min trials in which we assessed investigation of water droplets (trials 1–3), diluted urine from an unfamiliar same-sex mouse (trials 4–6), and diluted urine from a second unfamiliar same-sex mouse (trials 7–9) [44]. Urine was collected from adult males and females that were not in the study. Mice were firmly gripped on the scruff of the neck and urine was collected into centrifuge tubes and frozen at −20°C. Individual samples were thawed and diluted 1 to 10 in water before testing. Ten minutes before testing, cagemates of the mouse to be tested were removed from the homecage. A drop of 10 µl of distilled water was pipetted onto the center of a glass slide which was then transferred into the cage for a 2 min trial. Slides were only used once per trial to control for the possibility that focal mice might mark the slide with urine (although this was not observed in this study). During each trial the amount of time spent investigating the drop was recorded. Two more trials with distilled water were then conducted with a one minute inter-trial interval. After the third trial with water, three additional trials were conducted with 10 µl of diluted urine placed on a slide. All mice were tested with diluted urine samples from a same-sex mouse. The same sample was used for each of the next three trials (4–6). To test whether mice could discriminate between two odors each mouse was then tested with urine samples from a second same-sex unfamiliar mouse (trials 7–9). Immediately after testing the cagemates were returned to the home cage, and only one mouse per cage was tested per day.

We tested mice in the open field test because the presence of the wire cage in the social interaction tests alters activity patterns and is not strictly speaking an open field. Each mouse was placed in a large arena (89×63×60 cm) for 10 min and was tracked using a video tracking system. The light-dark box consisted of a 89×63×60 cm arena divided in half with a partition containing a small opening (7.6×7.6 cm). Half of the box was covered completely with a thick cloth. Tests were 5 min long and conducted in the light phase.

#### Corticosterone Assay

Corticosterone was assayed using an I^125^ labeled radioimmunoassay kit (MP Biomedicals, Solon, OH) that has been used previously with California mice [18], [45]. California mice have very high baseline corticosterone levels, so samples were diluted 1∶2000. The sensitivity of this assay is 25 ng/mL. The intra-assay coefficient of variation was 4.3%.

#### Data Analysis

Non-parametric Mann-Whitney and Wilcoxon tests were used to analyze data from habituation-dishabituation tests because of non-normal distribution of data. Non-parametric Mann-Whitney tests were also used to analyze corticosterone data. Two-way ANOVA was used to analyze data from open field and light-dark box tests as Q-Q plots indicated these data were normally distributed, and the variances between treatment groups were homogenous.

## Results

### Experiment 1

One day after the last episode of defeat, males spent more time interacting with the novel mouse (Table 1, F_1,41_ = 4.1, p<0.05) than females and no overall effects of stress or interaction were observed. However at four weeks, there was a significant sex x stress interaction on social interaction time (Table 1, Fig. 1C; F_1,41_ = 6.31, p<0.02). Stressed females spent significantly less time than control females interacting with the novel mouse (planned comparison, p<0.001) but there was no difference in males (planned comparison, p>0.58). Reduced social interaction responses in females persisted across different stages of the estrous cycle (Fig. 1D; sex x stress interaction F_2,41_ = 3.94, p<0.05). Both diestrus (planned comparison, p<0.001) and proestrus/estrus (planned comparison, p<0.02) females exposed to social defeat spent significantly less time interacting with the target mouse than control females. There were no significant differences in time spent interacting with the empty cage at 24 hr or 4 weeks after social defeat (Table 1). There were no significant differences in time spent in the corner zones or total activity during either the 24 hr or 4 week tests (all p's>0.15).

**Table 1:** Behavioral data from social interaction tests with empty cage and a novel mouse (target).

	Male	Male	Female	Female
	Control	Stress	Control	Stress
*24 hr after last defeat*				
Time in interaction zone (empty)	84.3±10.2	87.9±10.3	66.8±6.8	70.3±8.1
Time in interaction zone (target)	132.4±10+++	113.7±11+	103.5±11.2++	89.4±10.7
Time in corners (empty)	6.3±1.8	4.1±1.5	8.3±2.2	10.1±3.7
Time in corners (target)	2.6±1.0	6.0±2.0	8.46±2.8	7.0±2.2
Total distance (empty)	27.4±3.8	25.7±2.0	30.0±2.5	27.4±1.5
Total distance (taget)	23.5±4.1	24.3±4.5	26±2.6	27.3±2.4
*4 weeks after last defeat*				
Time in interaction zone (empty)	100.3±7.6	102.4±8.6	104±10.9	80.5±9.2
Time in interaction zone (target)	137.8±9.8++	128.7±13+	152±8.2+++	89.9±9.7**
Time in corners (empty)	2.6±0.9	4.28±1.6	4.3±1.4	6.7±1.3
Time in corners (target)	2.4±0.9	4.8±2.2	3.6±2.3	5.6±1.7
Total distance (empty)	29.9±4.4	25.1±2.7	27.3±4.1	29.8±1.8
Total distance (taget)	26.8±5.2	24.9±4.5	20.4±4	27±2.5

All times in sec, all distances in m.

+ p<0.05,

++ p<0.01,

+++ p<0.001 paired t-test with empty cage trial (within group).

** p<0.01, planned comparison control vs. stress following significant sex x stress interaction. All data are mean±s.e.

**Figure 4:**
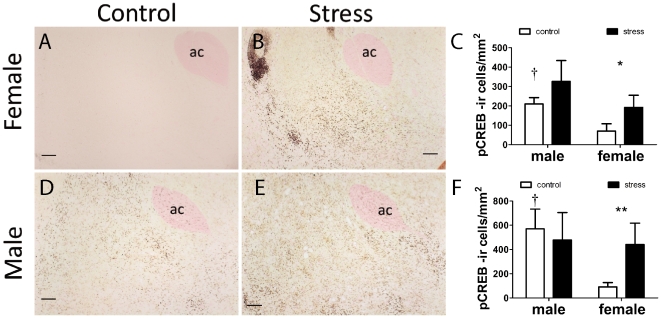
Immunostaining for phosphorylated CREB in female (A, B, C) and male (D,E, F) California mice after social interaction tests. Mice were exposed to three control or social defeat episodes. Social defeat increased the number of pCREB positive cells in females but not males in the NAc shell (C) and core (F). Control males generally had higher pCREB cell counts than control females. † Mann-Whitney sex difference in controls p<0.05, *, **, Mann-Whitney effect of stress p<0.05, p<0.01 respectively. All data are mean±s.e. Anterior commissure, ac. Scale bars = 100 µm.

**Figure 5:**
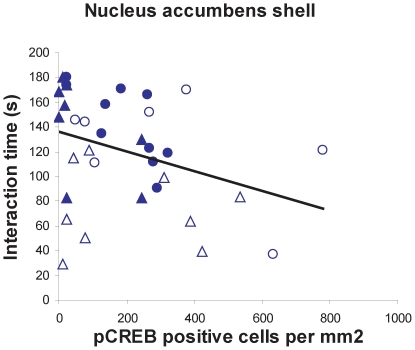
Correlation between pCREB positive cells in NAc shell and interaction time with a novel target mouse. Filled circles (control males), open circles (stressed males), filled triangles (control females), open triangles (stressed females). Spearman ρ = −0.37, p<0.05.

**Figure 6:**
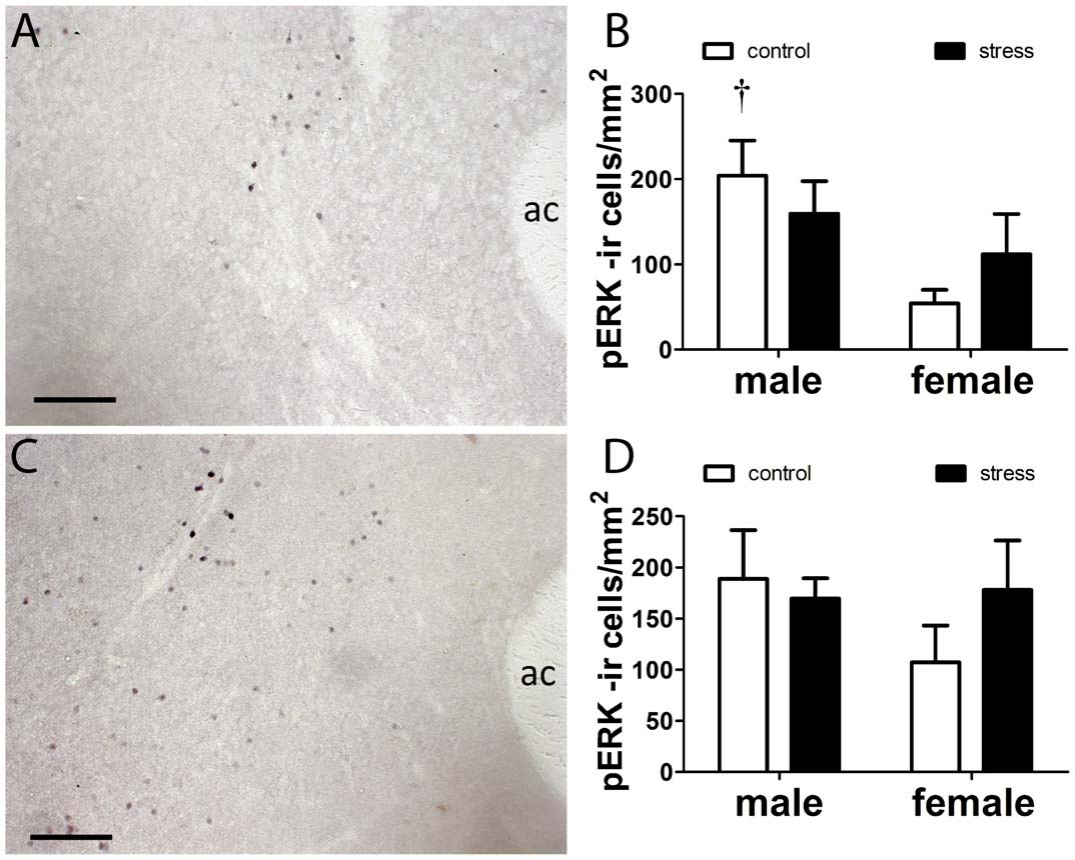
Immunostaining for phosphorylated ERK in female (A) and male (C) NAc. Males had more pERK positive cells than females in the NAc shell (B) but not in the NAc core (D). † Mann-Whitney sex difference in controls p<0.05. All data are mean±s.e. Anterior commissure, ac. Scale bars = 100 µm.

In the NAc females exposed to defeat had more pCREB positive cells in the shell (Fig. 4C, Mann-Whitney U, p<0.05) and core (Fig. 4F, p<0.05) immediately following social interaction testing compared to control females. In males there was no significant effect of stress on pCREB positive cells in either the NAc shell (Fig. 4C) or NAc core (Fig. 4F). Control males had more pCREB positive cells than control females in the NAc shell (Fig. 4C, Mann-Whitney, p<0.01). Across all mice, pCREB positive cells in the shell were negatively correlated with time spent interacting with the target mouse (Fig. 5, Spearman ρ = −0.37, p<0.05). There were no significant correlations between time spent interacting with the target mouse and pCREB positive cells in the core (overall or within males or females). There were no effects of stress on pERK positive cells in the NAc (Fig. 6), but control males had more pERK positive cells than control females in the NAc shell (Fig. 6B, Mann-Whitney, p<0.05).

In the amygdala, pCREB cell counts were not closely associated with patterns of social interaction behavior (Table 2). Control males had higher cell counts than control females in the dorsal MEA, BLA, and CEA but there were no effects of stress. Although cell count data in the amygdala and PVN were not correlated with behavior in the social interaction test, there were interesting relationships between nuclei. In females there were positive correlations between the NAc shell and CEA (Spearman ρ = 0.51, p<0.05) and between NAc shell and BLA (ρ = 0.54, p<0.05). These relationships were absent in males (p's>0.2). Females exposed to social defeat had more pERK positive cells in the PVN compared to control females (Table 2, Mann-Whitney, p<0.05). No differences were observed in males. Overall, there was a nonsignificant trend for a negative correlation between pERK positive cell counts in the PVN and time spent interacting with the novel mouse (Spearman ρ = −0.33, p = 0.06). There were no significant differences in pCREB immunostaining in the BNST.

**Table 2:** Cell counts for pCREB and pERK positive cells immediately after social interaction tests.

	Male	Male	Female	Female
	Control	Stress	Control	Stress
*pCREB*				
dorsomedial BNST	344±88	132±142	355±142	217±109
dorsolateral BNST	395±71	342±125	464±207	235±66
ventromedial BNST	1,094±262	481±99	1,036±295	975±306
paraventricular nucleus	814±137	580±60	897±144	657±130
ventromedial amygdala	1,117±90	967±340	995±362	956±261
dorsomedial amygdala	570±142†	506±161†	233±99	253±167
central nucleus amygdala	1,874±154	1,386±243	937±183	1,069±138
basolateral amygdala	272±118†	172±76	37±11	57±16
*pERK*				
dorsomedial BNST	69±12	38±15	38±14	47±21
dorsolateral BNST	4±4	5±3	9±8	5±2
ventromedial BNST	174±42†	57±17	105±54	132±30
paraventricular nucleus	961±255	853±191	740±267*	1,756±360
ventral medial amygdala	176±81	141±28	158±54	218±42
dorsal medial amygdala	46±24	32±13	39±27	30±10
central nucleus amygdala	103±30	52±24	118±80	141±57
basolateral amygdala	163±49	123±32	117±75	85±34

† sex difference within treatment group p<0.05 Mann-Whitney test.

*effect of stress within sex p<0.05 Mann-Whitney test.

All data are mean±s.e.

Immediately after social interaction testing, females had higher corticosterone levels than males (Fig. 1E, Mann-Whitney U, p = 0.03), but there was no effect of stress or interaction. Corticosterone levels were not correlated with social interaction time in either males or females.

### Experiment 2

During episodes of social defeat both males (mean±s.e. offensive attacks, 8.1±0.4) and females (7.0±0.4) were exposed to offensive attacks, although the number of attacks was higher in males (Repeated measures ANOVA F_1,18_ = 4.66, p = 0.046).

**Figure 7:**
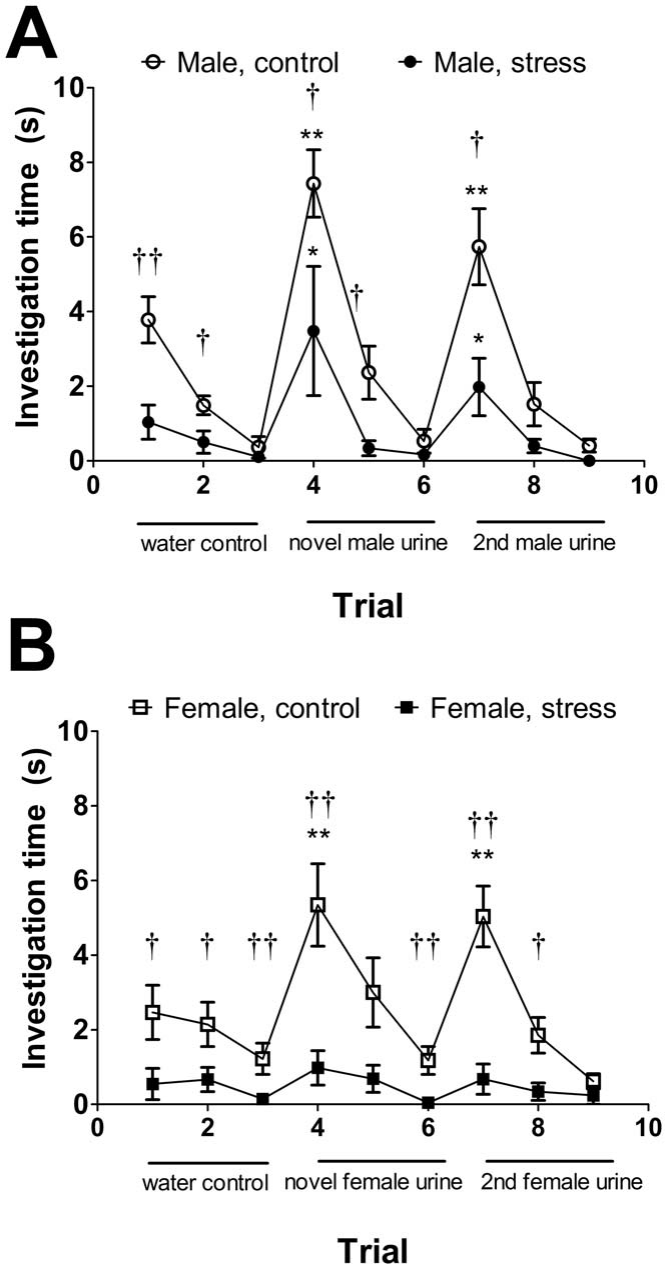
Effects of social defeat in the habituation-dishabituation test. Social defeat reduced time spent investigating a glass slide with a water droplet (trials 1–3) in both males (A) and females (B). In males defeat reduced, but did not eliminate investigation of novel male odors (trial 4 and 7). Females exposed to defeat did not show a significant increase in investigation time of novel female odors (trials 4 and 7). †, †† Mann-Whitney effect of stress p<0.05 and p<0.01 respectively. *, ** Wilcoxon test versus previous trial (trial 3 vs. 4 or 6 vs. 7) p<0.05, p<0.01 respectively. All data are mean±s.e.

In habituation-dishabituation tests, both control and stressed males (Fig. 7a) showed a significant increase in time spent investigating urine from unfamiliar males. In contrast, only control females showed a significant increase in time spent investigating urine from unfamiliar females whereas stressed females did not (Fig. 7b). Social defeat, reduced the amount of time both males and females investigated both social and non social odors (water controls), suggesting that social defeat may induce an aversion to novel objects within the home cage.

**Figure 8:**
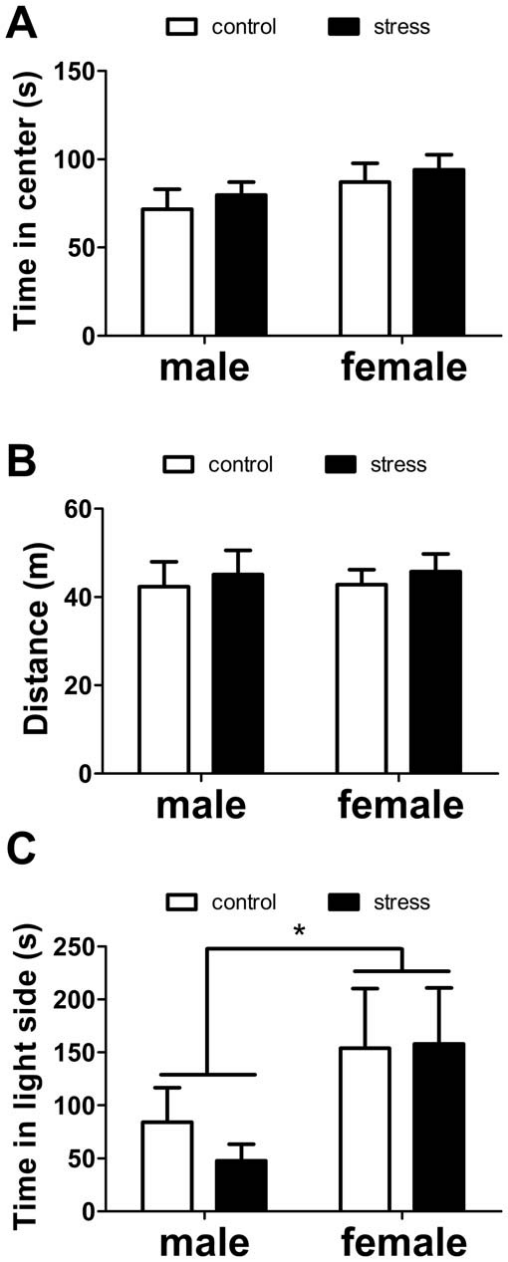
Effects of social defeat on time spent in the center (A) and total activity (B) in the open field test and time spent on the light side of the light-dark box test (C). * main effect of sex p<0.05. All data are mean±s.e.

There were no main effects of sex or stress, or interactions on time spent in the center of the open field or total activity (all p's>0.28, Fig. 8). In the light-dark box, females spent significantly more time in the light side compared to males (Fig. 8, F_1,33_ = 4.65, p<0.05). There were no differences in the latency to enter the light side or the number of entries (all p's>0.13).

**Figure 9:**
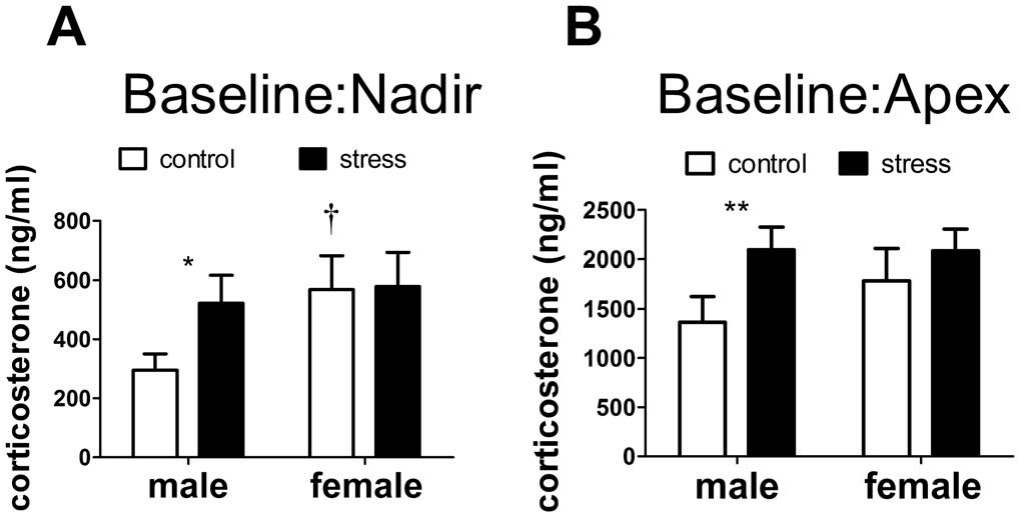
Effects of social defeat on corticosterone. Social defeat increased baseline corticosterone in males but not females during both the inactive (A) and active (B) phases. *, ** Mann-Whitney effect of stress p<0.05, p<0.01 respectively. † Mann-Whitney sex difference in controls p<0.05. All data are mean±s.e.

During the light (inactive, nadir) phase, stressed males had higher baseline corticosterone levels than control males (Fig. 9a, Mann-Whitney U, p<0.05) but there was no effect of stress on females. Control females also had higher corticosterone than control males (Mann-Whitney U, p<0.05) in the light phase. In the dark (active, apex) phase stressed males had higher corticosterone levels than control males (Fig. 9b, Mann-Whitney U, p<0.05) and there was no effect of stress in females. There was no significant difference in corticosterone between control males and females during the dark phase.

## Discussion

Although the social defeat model robustly induces behavioral responses related to mood disorders, its use has been constrained almost entirely to males. Using monogamous California mice we demonstrate for the first time that social defeat can induce a long lasting reduction in social interaction behavior in females. This behavioral change was not associated with changes in general activity or exploratory behavior, suggesting that the effects of social defeat in female California mice are relatively specific to social contexts. Social interaction behavior was negatively correlated with pCREB expression in the NAc shell, a relationship that is consistent with previous studies linking the mesolimbic dopamine system and reduced social interaction behavior in male rodents [8], [21], [46] as well as findings of altered striatal dopamine function in humans with social phobias [47], [48](but see [49]). Our results suggest that the mesolimbic dopamine system could be more sensitive to social stress in females versus males.

### Effects of Defeat on Social Interaction

In experiment 1 the effect of social defeat on social interaction behavior in females was stronger after 4 weeks following defeat versus one day after. This suggests that the effects of social defeat may grow stronger over time, possibly due to long term changes in gene expression or synaptic remodeling. To our knowledge, this is the first demonstration of a long lasting reduction in social interaction behavior by defeat in a female mammal. There was little evidence that females exposed to defeat increased time spent in the corners, as has been reported in studies on male domestic mice [8]. This could reflect reduced motivation to engage in social stimuli as opposed to increasing motivation to avoid social stimuli. An alternative possibility is that the relatively large arena that we used allowed mice to avoid the social stimulus without entering the corners. There were no effects of social defeat on time spent interacting with the empty cage, indicating that changes in behavior were specific to social stimuli. In the social interaction test mice were placed in a novel environment, which is known to be anxiogenic [50]. Social interaction testing has become prevalent in various forms [8], [51], [52], [53], and the vast majority of studies are conducted in a novel environment. However, the potential anxiogenic effects of a novel environment are rarely considered.

We used the habituation-dishabituation tests to examine responses to social odors in a familiar environment. These results generally supported our findings in the social interaction tests, as defeat had a greater impact on the investigation of social odors in females versus males. However, male and female mice exposed to defeat spent less time investigating glass slides with water (a relatively benign stimulus) than control mice. This suggests that defeat may induce an aversion to novelty, at least in a familiar environment. It is possible that an aversion to novelty could affect measurements of social interaction. However, stressed males still responded to same sex social odors whereas stressed females did not. An additional possibility is that females exposed to defeat may have developed reduced sensitivity for detecting social stimuli. In domestic mice, social isolation rearing does not block investigation of social odors but inhibits social learning and recognition [54]. The effects of social defeat on social learning or recognition in female California mice are still unclear.

All mice were group housed with familiar cagemates throughout the study. Group housing blunts the effects of social defeat in male rats [55], an effect that could be due to positive social interactions. However group housing did not have this effect in female California mice. In rats it is possible that aggressive interactions among cagemates could mediate the effects of stress on behavior. For example, aggressive behavior directed at other individuals following stress has been observed in several species [56], [57], and has been hypothesized to be a coping strategy. Currently little is known about home cage social interactions among rodents immediately following stressful experiences.

### Effects of Defeat on Brain Responses to a Novel Target Mouse

Previous research in male domestic mice demonstrated that the NAc is an essential nucleus inducing reduced social interaction behavior following social defeat [8], [21]. In addition, two recent studies reported that deep brain stimulation of the NAc reduces ratings of depression and anxiety in at least some patients for whom other standard treatments have been ineffective [58], [59]. In female California mice exposed to social defeat, we observed elevated pCREB immunostaining in the NAc shell and core after social interaction tests compared to control females. Although there were no significant effects of stress on pCREB immunostaining in males, there was intriguing variability among stressed males. In particular, two males exposed to defeat had almost double the number of pCREB positive cells than next highest male (Fig. 5). These males also had relatively low social interaction scores and contributed to (but were not solely responsible for) the negative correlation between pCREB immunostaining in the NAc shell and social interaction time. These observations are consistent with the hypothesis that increased activity in the NAc can inhibit social interaction. Studies of hundreds of C57Bl6 mice have classified males as susceptible or unsuceptible to social defeat based on variation in behavioral responses in a social interaction test [21]. Our results suggest that similar variation may exist in male California mice. We also observed that control males had higher pCREB and pERK cell counts in the NAc shell than control females. Intriguingly, many studies in rodents examining biochemical function in the NAc have observed a lack of sex differences [60], [61], [62]. However, most of these studies focus on baseline biochemical function as opposed to responses to an acute stimulus. For example, male rats have a sustained upregulation in pCREB expression in response to cocaine compared to females [61]. Thus sex differences in NAc function may be context dependent, and difficult to observe in the absence of a motivating stimulus such as a drug or novel individual.

Studies in male hamsters have identified the BNST and several nuclei of the amygdala as important brain regions mediating behavioral responses to social defeat [63], [64], [65]. In the present study we observed no effects of social defeat on pCREB or pERK cell counts in these nuclei. Social defeat may indeed alter activity of these nuclei, but these changes may not be reflected in pCREB or pERK expression in response to a social stimulus. An additional possibility is that the BNST and amygdalar nuclei have a more important role immediately after social defeat [66]. We observed that males had higher pCREB cell counts in the dorsal MEA and BLA compared to females. Cell counts in these nuclei were not correlated with behavior in the social interaction tests, so the function of these sex differences is still unclear. Future functional studies are needed to test whether the BNST and amygdalar nuclei mediate the effect of defeat on social interaction responses.

### Effects of Defeat on Corticosterone Levels

Several studies have observed that a subset of patients with depression have elevated cortisol levels [67], particularly in the evening nadir [68], [69], [70]. Although we observed reduced social interaction behavior in female California mice, elevated baseline corticosterone levels were not observed in females. It is possible that stress-induced decreases in social interaction are induced in the absence of an increase in baseline glucocorticoids. However, this does not rule out glucocorticoids as a contributing factor. As has been reported in numerous studies on rats [71], [72], [73], we observed that control females had higher baseline corticosterone levels than control males during the light phase. There is considerable evidence that gonadal hormones contribute to this sex difference in glucocorticoid secretion [74], and increased glucocorticoid reactivity has been hypothesized as a contributing risk factor for stress-induced diseases, including mental disorders [75]. In experiment 1, both stressed and control female California mice had higher corticosterone levels than males immediately following social interaction tests. Although there was no effect of social defeat in females, this could be due in part to a ceiling effect. It is also possible that females exposed to social defeat have a slower recovery profile than control females. This hypothesis is supported by our observation of increased pERK cell counts in the PVN of females exposed to defeat. In follow-up experiments we have observed that ovariectomy diminishes corticosterone responses to social defeat, as well as ameliorating the effect of defeat on social interaction behavior (Trainor, Silva, Takahashi & Knoblauch unpublished), suggesting that ovarian hormones are involved in mediating sex differences in response to defeat. Our current findings suggest that female California mice (both control and stressed) have exaggerated corticosterone responses to novel same-sex mice in unfamiliar contexts, similar to what has been observed in familiar contexts [18]. It should be noted that the high corticosterone levels we observed in this study are consistent with observations by other lab groups studying California mice [41], [45], [76].

Intriguingly, social defeat increased baseline corticosterone levels in males during both the light and dark phases. This is despite the fact that few differences in behavior were observed between stressed and control males. One possibility is that only a subset of males exposed to social defeat show behavioral responses, similar to what has been observed in C57Bl6 mice. An alternative possibility is that males exposed to social defeat exhibit behavioral changes in contexts that were not examined in this study, such as the forced swim or tail suspension test. Further study is needed to fully resolve male California mouse behavioral responses to defeat.

### Summary

A major weakness of the social defeat model has been an inability to test hypotheses in females. This weakness was overcome by studying the California mouse, and we demonstrated for the first time that social defeat induces long lasting decreases in social interaction behavior in female but not male California mice. Our analyses of pCREB immunostaining suggest that the NAc shell could be an important locus mediating effects of social defeat on social interaction behavior. Social withdrawal is associated with many mental disorders, so a better understanding of the neurobiological mechanisms influencing social interaction behavior could be applicable to many contexts.
